# Δ^9^-THC and *N*-arachidonoyl glycine regulate BV-2 microglial morphology and cytokine release plasticity: implications for signaling at GPR18

**DOI:** 10.3389/fphar.2013.00162

**Published:** 2014-01-02

**Authors:** Douglas McHugh, Daniel Roskowski, Sisi Xie, Heather B. Bradshaw

**Affiliations:** ^1^Department of Psychological and Brain Sciences, Indiana UniversityBloomington, IN, USA; ^2^Frank H. Netter MD School of Medicine, Quinnipiac UniversityNorth Haven, CT, USA

**Keywords:** Δ^9^-THC, NAGly, microglia, BV-2, phenotype, cytokine, morphology, GPR18

## Abstract

Microglial cells are extremely plastic and undergo a variety of CNS-prompted shape changes relative to their location and current role. Signaling molecules from neurons also regulate microglial cytokine production. Neurons are known to employ the endogenous cannabinoid system to communicate with other cells of the CNS. *N*-arachidonoyl glycine (NAGly) and Δ^9^-tetrahydrocannabinol (Δ^9^-THC) signaling via GPR18 has been introduced as an important new target in microglial–neuronal communication. Our hypothesis is that endogenous NAGly-GPR18 signaling regulates phenotypic shape and cytokine production in microglia, and is mimicked by Δ^9^-THC in the BV-2 microglia model system. BV-2 microglia were exposed to NAGly and Δ^9^-THC or Vh for 12 h, which resulted in significant differences in the cell morphologies expressed. Cannabidiol (CBD) was effective at antagonizing the effects of both NAGly and Δ^9^-THC. Using ELISA-based microarrays, BV-2 microglia were exposed to NAGly and Δ^9^-THC or Vh for 3 h and the presence of 40 cytokines in the culture media quantified. Production of Axl, CD40, IGF-I, OPN, and Pro-MMP-9 were significantly altered by NAGly and Δ^9^-THC, and antagonized by CBD. These data add to an emerging profile that emphasizes NAGly as a component of an endogenous system present in the CNS that tightly integrates microglial proliferation, recruitment, and adhesion with neuron–glia interactivity and tissue remodeling.

## INTRODUCTION

Microglial cells are extremely plastic and undergo a variety of shape changes relative to their location and current role (Block et al., 2007; Colton and Wilcock, 2010). *Ramified* microglia possess a characteristic form composed of long branching processes that are very sensitive to alterations in the local microenvironment (Fetler and Amigorena, 2005; Raivich, 2005). *Amoeboid* microglia scavenge CNS plaque and foreign agents and mediate cytotoxic and inflammatory signaling (Gordon, 2003; Fetler and Amigorena, 2005; Raivich, 2005; Garden and Moller, 2006). Microglia have a significant impact on overall CNS stability and may act as an essential link in the interaction of cytokines with neurons (Zhang et al., 2003). In both developmental and post-developmental contexts, microglia discriminately engulf and eliminate dead or dying neurons. They are especially prevalent during rewiring of the brain when there are large amounts of extracellular apoptotic debris to remove.

These processes must be tightly controlled in order to sustain the least possible collateral damage to adjacent neurons. Indeed, microglial activity is not only orchestrated to exacting standards, but the coupling between the death of neurons and their degradation by microglia is both striking and swift. This remarkable relationship suggests a fast-acting communication between neurons and microglia, such that the microglia are forewarned of the specific task (i.e., apoptosis, infection, or damage). Once the phagocytic task of amoeboid microglia is complete a return to the *resting* (i.e., multi-branched, surveillant) form is usually observed. Yet, microglia are also implicated in virtually all CNS neuropathological processes, where they become highly reactive to dying neurons and provoke sustained secondary neurotoxicity (Carson, 2002; Eikelenboom et al., 2002; Streit, 2002; Danton and Dietrich, 2003; van Rossum and Hanisch, 2004). This extreme dichotomy of behavior underscores the importance of understanding the specific signaling systems that both recruit and instruct microglia to adapt their phenotype selectively in response to damaged/dying/apoptotic neurons, and which are foundational to our ability to monitor and manage dysregulated microglial activity.

Neurons are known to employ the endogenous cannabinoid system to communicate with other cells of the CNS. Walter et al. (2003) reported the involvement of the endogenous cannabinoid signaling in recruiting microglia toward dying neurons *in vitro*. Recently the pharmacology of *N*-arachidonoyl glycine (NAGly), various cannabinoids [most notably Δ^9^-tetrahydrocannabinol (Δ^9^-THC)] and other signaling ligands at GPR18 receptors has been described. During the same timeframe, NAGly-GPR18 signaling was also introduced as an important new target in microglial–neuronal communication (see McHugh, 2012; McHugh et al., 2012a,b for review). NAGly is synthesized in the CNS primarily from AEA via a fatty acid amide hydrolase (FAAH) dependent pathway and can be prevented by URB597, an irreversible inhibitor of FAAH (Bradshaw et al., 2009). It is ineffective as an agonist at either CB_1_ or CB_2_ receptors despite the obvious structural overlap with AEA (Járai et al., 1999; Begg et al., 2005; Mackie and Stella, 2006). Instead, NAGly acts as a high affinity ligand for the G_i/o_-coupled GPCR GPR18 (Kohno et al., 2006; McHugh et al., 2010, 2012a,b; McHugh, 2012) and a partial agonist of G_q/11_-coupled GPR92 receptors (Oh et al., 2008). Δ^9^-THC is a well-established agonist at G_i/o_-coupled cannabinoid CB_1_ and CB_2_ receptors (for review, see Pertwee et al., 2010). More recent investigations have raised the prospect of Δ^9^-THC signaling via non-CB_1_ and non-CB_2_ GPCRs, including GPR55 and GPR18, although the current pharmacology of GPR55 is complicated and inconsistent (Pertwee et al., 2010). Here, we explore the hypothesis that NAGly–GPR18 signaling regulates morphological and cytokine signaling plasticity, which is mimicked by Δ^9^-THC in the BV-2 microglia model system.

## MATERIALS AND METHODS

### TEST COMPOUNDS

Cannabinoids and related lipids are practically stable during storage, with concentration changes attributed to oxidation, temperature effects, and lipophilic binding. The lipid compounds tested in this study were prepared in 100% DMSO to a stock concentration of 100 mM, aliquoted and kept at -20°C. Fresh aliquots were used for each independent experiment. Compounds were serially diluted to achieve the desired final working concentrations, where each contained 0.1% DMSO as vehicle. NAGly was purchased from Cayman Chemicals (Farmingdale, NY, USA), lipopolysaccharide (LPS), and phorbol 12- myristate 13- acetate (PMA) from Sigma-Aldritch (St. Louis, MO, USA). Δ^9^-THC and Cannabidiol (CBD) were provided by Dr. Ken Mackie, Indiana University.

### CELLS IN CULTURE

The mouse microglial cell line BV-2 (a gift from Dr. N. Stella; University of Washington, Seattle), which was originally generated by immortalizing primary microglia (Blasi et al., 1990), were grown in high glucose DMEM (Gibco, USA) supplemented with FBS (5%), penicillin (100 units/ml), streptomycin (100 μg/ml), and L-glutamine (0.292 mg/ml). Cells were passaged every 2–3 days for a maximum of 30 passages. Media was replaced with serum-free Ham’s F-12 phenol red-free media (Gibco, USA), supplemented with penicillin (100 units/ml), streptomycin (100 μg/ml) and L-glutamine (0.292 mg/ml), 16 h prior to the start of experiments.

### BV-2 MORPHOLOGY EXPERIMENTS

**FIGURE 1:**
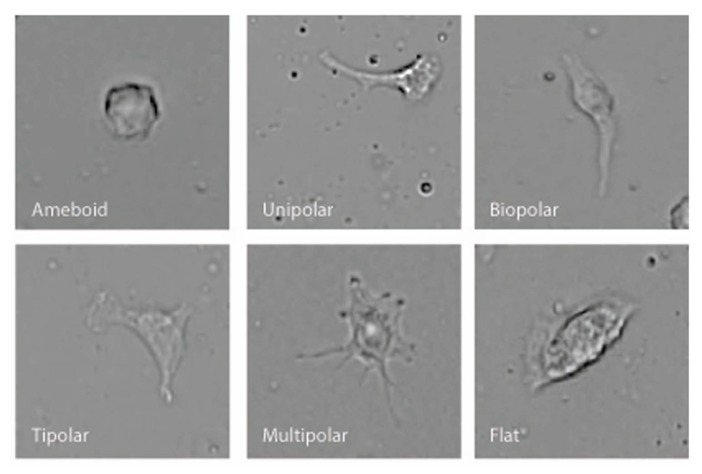
**Examples of the six different BV-2 morphological shapes observed and categorized**.

BV-2 microglia were plated using serum-free Ham’s F-12 phenol red-free media on 25 mm × 75 mm glass microscope slides that had been pre-coated for 1 h with 1 μg/ml poly-D-lysine. A coated microscope slide was placed in a 10 cm Petri dish then 1 ml of cell suspension (5 × 10^5^ cells/ml) was placed onto the center of the slide using a Pasteur pipette. Then an additional 11 ml of the Ham’s F-12 media was added to the dish together with Vh or test compound. Cells were incubated for 12 h (5% CO_2_ atmosphere at 37°C) with Vh control (0.1% DMSO), 10 nM NAGly, 10 nM Δ^9^-THC, 100 nM CBD, 10 nM NAGly + 100 nM CBD, or 10 nM Δ^9^-THC + 100 nM CBD. 10 nM PMA was used as positive control. Each condition was tested in triplicate per individual experiment. The media was removed and replaced for 20 m with 12 ml of cytoskeleton buffer with sucrose and formaldehyde. This buffer consisted of 10 mM 2-*N*-morpholino ethanesulfonic acid (MES), 138 mM KCl, 3 mM MgCl_2_, 2 mM EGTA in 1 L of H_2_O, with its pH adjusted to 6.1. It was stored at 4°C in between use. 0.32 M sucrose was added to a 50 ml aliquot of the solution prior to each experiment and thoroughly vortexed. Next formaldehyde was added to the aliquot to achieve a concentration of 3.7%. Following the 20 m incubation, the slides were then washed with phosphate-buffered saline (PBS) three times. Next, permount was pipetted onto the slide and a glass coverslip placed on top. Slides were then put in a dark drawer, where they were protected from light, and left to dry for at least 24 h. Images from 10 random fields of view were collected at x20 magnification using a Nikon 80i light microscope from each microscope slide. Cell morphology was recorded and categorized by multiple investigators who were blind to the treatment condition into six categories: amoeboid, unipolar, bipolar, tripolar, multipolar, and flat. This amounted to 200+ cells per individual treatment replicate. Categorization was based on the number of processes that branched directly off the cell body. A cell was considered to be unipolar if it has one process, bipolar two, tripolar three, and multipolar four or more. The amoeboid and flat categories had zero processes. To distinguish between these, cells that were overtly three-dimensional and irregularly rounded were classed as amoeboid; while those with their cytoplasm spread over a larger area, with wavy edges and little three-dimensional depth were classed as flat (**Figure 1**).

### BV-2 CYTOKINE RELEASE EXPERIMENTS

~ 90% confluent BV-2 cells were incubated for 3 h (5% CO_2_ atmosphere at 37°C) in 3 ml serum-free Ham’s F-12 phenol red-free media with Vh control (0.1% DMSO), 10 nM and 100 nM NAGly, 10 and 100 nM Δ^9^-THC, 100 nM CBD, 10 nM NAGly + 100 nM CBD, and 100 nM Δ^9^-THC + 100 nM CBD. 10 nM PMA was used as positive control. 1 μg/ml LPS was used as positive control. After the incubation, the culture media was carefully removed (to minimize transferring any cells) and placed in 15 ml centrifuge tubes. Tubes were left undisturbed on the bench for 10 min to allow any cell debris to settle to the bottom. 100 μl of culture media was then collected and the presence of 40 cytokines quantified using Quantibody Mouse Cytokine Array 4 (RayBiotech, Inc.), Odyssey blocking buffer (Li-Cor #927-40000), Li-Cor IRDye 800CW Streptavidin (#926-32230), and Odyssey infra-red imager (Li-Cor Biosciences). The Quantibody kit is a multiplexed, sandwich ELISA-based array that enables researchers to accurately determine the concentration of multiple cytokines simultaneously. Odyssey settings were: resolution, 21 μm; quality, highest; focal offset, 1.0 mm; and intensity, 10 for the 800 nm channel (the 700 nm channel was not required). The cytokines tested by the array were as follows: AR, Axl, CD27L, CD30T, CD40, CXCL16, EGF, E-selectin, Fractalkine, GITR,HGF, IGFBP-2, IGFBP-3, IGFBP-5, IGFBP-6, IGF-I, IL-12p70, IL-17E, IL-17F, IL-1ra, IL-2 Rα, IL-20, IL-23, IL-28, I-TAC, MDC, MIP-2, MIP-3α, OPN, OPG, Prolactin, Pro-matrix metallopeptidase-9 (Pro-MMP-9), P-selectin, Resistin, SCF, SDF-1α, TPO, VCAM-1, VEGF, and VEGF-D.

## ANALYSIS OF DATA

All data are expressed as means ± s.e.mean and *n *= number of independent experiments. Statistical analyses were performed with GraphPad Prism 4 or SPSS. One-way ANOVA (*p* < 0.05) with LSD *post hoc* test was run to determine significance between treatment conditions.

## RESULTS

### EFFECTS OF Δ^9^-THC AND NAGly ON BV-2 MICROGLIAL MORPHOLOGY

**FIGURE 2:**
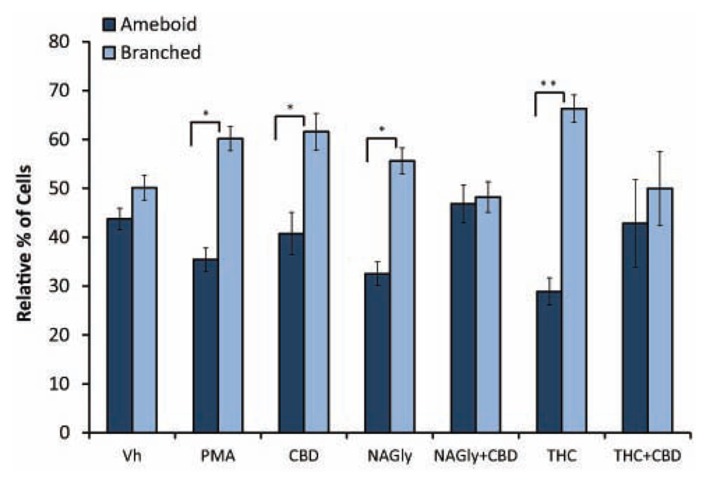
**Amoeboid versus branched phenotypes. ** Vehicle (0.1% DMS0), 10 nM PMA, 10 nM NAGly, 100 nM CBD, 10 nM NAGly + 100 nM CBD, 10 nM Δ^9^-THC, 10 nM Δ^9^-THC + 100 nM CBD. *n* = 5–15. *p < 0.05, **p < 0.01 (One-way ANOVA).

Microglial cells are extremely plastic and exist in a variety of morphologies, including: amoeboid, unipolar, bipolar, tripolar, multipolar, and flat, which are illustrated in **Figure 1**. At rest, BV2 microglia display primarily an amoeboid morphology (44%) with varying degrees of branched morphologies (**Table 1**). Data here replicated previous findings that 10 nM PMA induced a shift in morphology from one that was round and amoeboid to a non-round form with cytoplasmic extensions (**Table 1**). Likewise, 10 nM NAGly and 10 nM Δ^9^-THC significantly reduced the relative percentage of BV-2 microglia that manifest the amoeboid phenotype; instead, prompting them to adopt a non-round, branched morphology with cytoplasmic extensions, most of which was classified as being bipolar in shape (**Table 1**). Other than a small but significant decrease in the relative percentage of unipolar BV-2 microglia in the presence of 10 nM Δ^9^-THC + 100 nM CBD, Δ^9^-THC and NAGly produced non-significant fluctuations in the unipolar, tripolar, multipolar, or flat categories (**Table 1**). However, the morphologic phenotypic shift evoked by 10 nM NAGly and 10 nM Δ^9^-THC was antagonized by 100 nM CBD, which is in keeping with our previous reports of GPR18 pharmacology (McHugh et al., 2010, 2012a,b; McHugh, 2012). 100 nM CBD alone had no significant effects on BV-2 microglial shape (**Table 1**). When all branched morphologies are combined and compared to those cells in the amoeboid morphology, it is clear that there is an profound effect on the branched verses non-branched phenotype in the presence of Δ^9^-THC and NAGly and that this is reversed with CBD (**Figure 2**).

**Table 1:** Percentage of BV2 cell morphologies with vehicle and six treatment groups.

	Ameboid	Unipolar	Biopolar	Tripolar	Multipolar	Flat
Vehicle	43.75 ± 2.2	17.31 ± 1.2	19.81 ± 1.8	6.20 ± 0.8	6.80 ± 1.0	4.54 ± 0.8
PMA	35.44 ± 2.4^*^	19.59 ± 1.3	24.29 ± 2.0	6.85 ± 1.2	9.43 ± 2.0	4.33 ± 0.5
CBD	40.72 ± 4.3	18.82 ± 1.9	22.08 ± 3.0	5.85 ± 1.6	10.49 ± 2.0	4.13 ± 0.7
NAGly	32.53 ± 2.4*	18.40 ± 1.5	25.84 ± 1.9*	6.81 ± 0.7	8.82 ± 1.6	5.93 ± 1.0
NAGly+CBD	46.84 ± 3.9****^‡^	17.42 ± 1.8	18.34 ± 2.1****^‡^	5.09 ± 0.9	7.37 ± 2.1	4.95 ± 1.6
THC	28.90 ± 2.8*	15.68 ± 1.7	33.57 ± 4.0*	6.45 ± 1.9	10.59 ± 2.5	4.81 ± 0.8
THC+CBD	42.82 ± 8.9^#^	11.89 ± 2.6*	23.61 ± 4.4	4.06 ± 1.7	10.41 ± 4.2	7.20 ± 2.1

* denotes a significant difference from vehicle values (*p* < 0.05). ****^‡^ denotes significant difference from NAGly values (*p* < 0.05). ****^#^ denotes significant difference from Δ^9^-THC values (*p* < 0.05).

### EFFECTS OF Δ^9^-THC AND NAGly ON BV-2 MICROGLIAL CYTOKINE RELEASE

**FIGURE 3:**
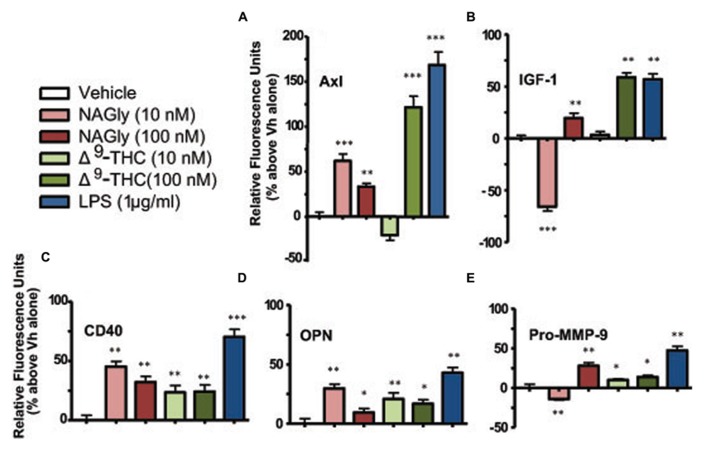
**Levels of cytokine production after 3 h treatment in BV-2 microglia.** **(A) **Axl, **(B)** IGF-1, **(C)** CD40, **(D)** OPN, **(E)** Pro-MMP-9. NAGly (10 nM and 100 nM) and Δ^9^-THC (10 nM and 100 nM). *n* = 4–5. *p < 0.05, **p < 0.01, ***p < 0.001 (One-way ANOVA).

Microglia signal and support numerous other cells by secreting a variety of signaling molecules and growth factors, including cytokines. Of the forty cytokines quantified by the Quantibody array, the production of five cytokines were significantly altered in BV-2 microglia in a concentration-dependent manner by NAGly and Δ^9^-THC. They were: Axl, CD40, IGF-I, OPN, and Pro-MMP-9 (**Figure 3**).

**FIGURE 4:**
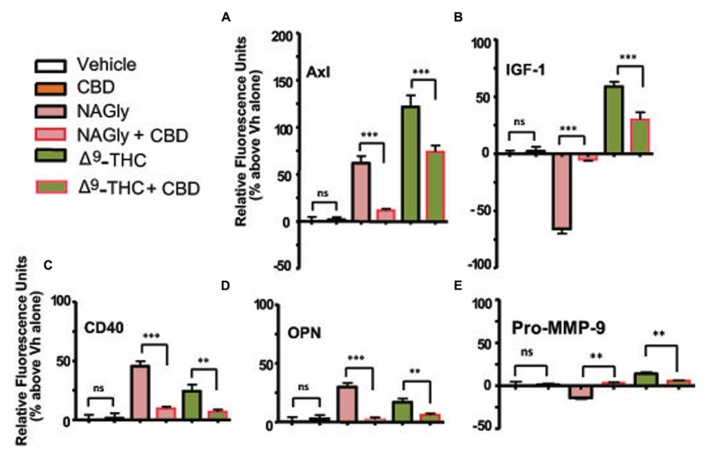
**Levels of cytokine production after 3 h treatment co-incubation with CBD in BV-2 microglia.** **(A)** Axl, **(B)** IGF-1, **(C)** CD40, **(D)** OPN, **(E)** Pro-MMP-9. Cytokine production by BV-2 microglia in response to NAGly (10 nM) and Δ^9^-THC (100 nM) ± CBD (100 nM) *n* = 4–5. **p < 0.01, ***p < 0.001 (One-way ANOVA).

Axl is a member of the tyrosine-protein kinase receptor (RTK) family and activated by the vitamin K-dependent protein, growth-arrest-specific 6 (Gas6; Mark et al., 1996). 10 nM and 100 nM concentrations of NAGly, 100 nM Δ^9^-THC and 1 μg/ml LPS caused a significant increase in the basal production of the cytokine Axl, while 10 nM Δ^9^-THC produced a significant reduction (**Figure 3A**). CD40 is a co-stimulatory protein and member of the TNF-receptor superfamily (Chatzigeorgious et al., 2009). 10 nM and 100 nM concentrations of NAGly and Δ^9^-THC together with 1 μg/ml LPS caused a significant increase in the basal production of the cytokine CD40 (**Figure 3B**). IGF-1 activates the Insulin-like growth factor 1 receptor (IGF1R), which is a receptor tyrosine kinase present on many cell types in many tissues (Jones and Clemmons, 1995). 100 nM Δ^9^-THC significantly increased basal IGF-1 levels, as did 1 μg/ml LPS, but 10 nM Δ^9^-THC had no effect. On the other hand, 10 nM NAGly produced a significant drop in IGF-1, while 100 nM NAGly produced a significant increase (**Figure 3C**). OPN is expressed in a range of immune cells, including macrophages, neutrophils, dendritic cells, and T and B cells (Mazzali et al., 2002). 10 nM and 100 nM concentrations of NAGly and Δ^9^-THC together with 1 μg/ml LPS caused a significant increase in the basal production of the cytokine OPN (**Figure 3D**). Pro-MMP-9 is the precursor form of MMP-9, which degrades type IV and V collagens (Toth et al., 2003). 10 nM and 100 nM Δ^9^-THC significantly increased basal Pro-MMP-9 levels, as did 1 μg/ml LPS and 100 nM NAGly. On the other hand, 10 nM NAGly produced a significant drop in Pro-MMP-9 (**Figure 3E**). Changes in cytokine production levels evoked by10 nM NAGly and 100 nM Δ^9^-THC for all five cytokines were significantly attenuated by 100 nM CBD (**Figures 4A–E**).

## DISCUSSION

Microglia play crucial roles in the development, on-going activity and regeneration of the CNS. For example, during CNS injury, microglia participate in inflammation and wound healing by migrating into damaged tissue, where they proliferate and act as phagocytic scavengers (Zhang et al., 2003). Microglia communicate with and support other cells by secreting a variety of signaling molecules and growth factors (Zhang et al., 2003). Microglia actively influence the environment of the CNS and may act as an essential link in the interaction of cytokines with neurons (Zhang et al., 2003). They also actively regulate the number of functional synapses in the CNS and glutamate receptor expression (Ji et al., 2013). This does not merely take place during development, but rather constitutes a dynamic, reversible, and continuous event in the mature brain. Hence, they are directly involved in the modulation of synaptic activity (Ji et al., 2013). In short, microglia are highly complex cells found in the CNS that exhibit multiple forms and functional specializations, and it has long been appreciated that microglial cells exist in extremely different cell shapes ranging from an amoeboid to a ramified phenotype. Indeed, microglial cells adapt their cell morphology to the meet the functional demands of their microenvironment: amoeboid, when they have to eliminate cell debris; ramified, when they have to keep their microenvironment under surveillance; branched cytoplasmic extensions, when they make direct contact with synaptic elements to modulate activity; and non-polarized, when they secrete cytokines and reactive oxygen or nitrogen species. Moreover, the CNS microenvironment also prompts microglia to adapt their cytokine release profile to meet functional demands (i.e., pro- versus anti-inflammatory). The mechanisms regulating these adaptations are poorly understood and prompt the question of what triggers cytological plasticity in microglia. Our previous research has shown that Δ^9^-THC and NAGly are full agonists at GPR18 with EC_50_ values in the nanomolar range, and that NAGly-GPR18 signaling drives potent microglia migration, proliferation as well as mitogen-activated protein kinase (MAPK) activation. Given this, we explored the hypothesis that Δ^9^-THC and NAGly, putatively also through GPR18 receptors, regulates morphological and cytokine signaling plasticity in BV-2 microglia.

### Δ^9^-THC AND NAGLY: MORPHOLOGICAL PLASTICITY

Our observations demonstrate that both Δ^9^-THC and NAGly influenced the shape of BV-2 microglia (**Table 1**). 10 nM concentrations of both molecules significantly reduced the proportion of cells that manifested the amoeboid phenotype by means of generally increasing the number of cytoplasmic processes branching off the microglial cell body. These morphological shifts were prevented by co-treatment with 100 nM CBD, which alone had no effects. (Pöttler et al., 2006) have reported that the addition of PMA decreased the number of amoeboid and increased the number of branched BV-2 microglia *in vitro*. Our results reproduced and confirmed their findings. A significant percentage of BV-2 microglia assumed a bipolar phenotype when exposed to Δ^9^-THC or NAGly. If the data is simplified by collapsing the shape categories into *amoeboid* versus *branched*, when exposed to Vh alone there is an almost equivalent amount of both phenotypes; however, the addition of PMA, NAGly, and Δ^9^-THC significantly shifted the percentage distribution in favor of *branched*, which was reversed by co-addition of CBD. This suggests that there may be a particular functional relevance to the *branched* morphology in the neuronal environment of the CNS. In support of this, recent reports state that non-amoeboid microglia contact active synapses, implying a cell-cell interaction between ramified microglia and neurons have also described how ramified microglia contribute to the protection of neurons from excitotoxicity (Tremblay et al., 2010; Fontainhas et al., 2011). Microglia are capable of adapting their cytoskeleton to adopt any of the six shapes categorized here. While this morphological plasticity is continuous in the sense that the changes are not binary or quantal, microglia do not necessarily have to progress from amoeboid to unipolar to bipolar to tripolar𠀦 etc. The different morphological stages of microglial activation and their functional consequences have not been fully ascertained in detail; this is an emerging field of interest to which our data contribute.

### Δ^9^-THC AND NAGly: CYTOKINE SIGNALING PLASTICITY

Cytokines are varied substances (e.g*.*, interferons, interleukins, growth factors, etc.) that are released by certain cells of the immune system and have an effect on other cells. The secretion profile of five cytokines (Axl, CD40, IGF-I, OPN, and Pro-MMP-9) was significantly altered in BV-2 microglia by Δ^9^-THC and NAGly; the changes were attenuated by CBD (100 nM). Axl is an enzyme of the RTK family and is involved in the transduction of signals from the extracellular matrix to the cytoplasm by binding growth factors like vitamin K-dependent protein growth-arrest-specific gene 6 (Gas6; Mark et al., 1996). Axl is involved in the mediation of cell aggregation by homotypic binding (Heide et al., 1998), which is an interaction between the same adhesion molecule expressed by different cell types (e.g*.*, neurons and microglia). Axl signaling plays a role in cell growth and survival in normal and cancer cells, including colon cancer and melanoma (Sensi et al., 2011). Interestingly, the first report to describe GPR18 arose unanticipated from expression studies by Gantz et al. (1997), who inadvertently isolated the GPCR from the human colonic cancer Colo 320DM cell line. While, Qin et al. (2010) reported that expression levels of GPR18 were not only constitutively active but also ~ 24-fold higher in human metastatic melanoma compared with benign nevi.

CD40 is a co-stimulatory protein and member of the TNF-receptor superfamily; it is found on antigen presenting cells and has been found to be essential in mediating a broad variety of immune and inflammatory responses (Chatzigeorgious et al., 2009). CD40 helps mediate the induction of potent microbicidal substances in glial cells, including reactive oxygen species and nitric oxide, leading to the destruction of ingested microbes (Cao et al., 2009). The interaction of this cytokine with TNF receptors have been found to be necessary for amyloid beta (Aβ)-induced microglial activation (Tan et al., 2002), and thus is thought to be an early event in Alzheimer’s disease pathogenesis. IGF-1 activates the IGF1R, which is a receptor tyrosine kinase present on many cell types in many tissues (Jones and Clemmons, 1995). It is a primary mediator of the effects of growth hormone and one of the most potent natural activators of the AKT signaling pathway (Gallagher et al., 2010). IGF-1 has growth-promoting effects on almost every cell in the body; it regulates cellular growth, development and DNA synthesis, especially in neuronal cells (Ueno et al., 2013). OPN is expressed in a range of immune cells, including macrophages, neutrophils, dendritic cells, and T and B cells (Mazzali et al., 2002). This cytokine is a chemoattractant that promotes cell recruitment to inflammatory sites. OPN also functions as an adhesion protein, involved in cell attachment and wound healing, and mediates cell activation, cell survival (by regulating apoptosis) and cytokine production (Mazzali et al., 2002). Proteins of the matrix metalloproteinase (MMP) family are secreted as inactive pro-MMP proteins and subsequently cleaved by extracellular proteinases (Toth et al., 2003). They are involved in the breakdown of extracellular matrix in normal physiological processes, such as embryonic development, reproduction, and tissue remodeling, as well as in disease processes, such as arthritis, intracerebral hemorrhage, and metastasis (Wang and Tsirka, 2005). MMP-9 degrades type IV and V collagens (Kähäri and Saariaho-Kere, 1997).

While there is general interest in, as well as reports in the literature and ongoing investigations into, the influence of cannabinoids on cytokine signaling, this is the first data regarding these five specific cytokines in BV-2 microglia. A synopsis of the overall functional roles of these five cytokines would include growth, adhesion, tissue remodeling and apoptotic regulation. Their modulation implies an important role for the underlying mechanism of action exploited here by Δ^9^-THC and NAGly. The effects observed were concentration-dependent, where the most extreme differences in response between 10 nM and 100 nM of NAGly and Δ^9^-THC were: decreased versus increased IGF-1 and Pro-MMP-9 basal levels for NAGly; and decreased/no change versus increased IGF-1 and Axl basal levels for Δ^9^-THC. Antagonism of Δ^9^-THC and NAGly effects by 100 nM CBD is in keeping with our previous reports of GPR18 pharmacology (McHugh et al., 2010, 2012a,b). Further investigation is needed to fully ascertain the involvement of GPR18 and to rule in or rule out other potentially relevant receptors (i.e., CB_1_, CB_2_, GPR92). A time course study leading up to and continuing beyond the time points used here would be beneficial in determining when and to what extent does maximal cytoplasmic branching and cytokine release induced by Δ^9^-THC and NAGly occur in the BV-2 cells. This would also help clarify the apparent switching of some of the cytokines from below basal to above basal levels (e.g., IGF-1 at 10 nM and 100 nM Δ^9^-THC and NAGly), as would tests with additional concentrations. Cytokine signaling is known to be complex, and often includes opposing responses (e.g., pro- versus anti-inflammatory) that are considered to be physiologically routine. Alternatively, ligand-receptors interactions at GPCRs are often subject to desensitization, which may explain the differential effects observed. As mentioned above, it is also possible that Δ^9^-THC and NAGly are acting via multiple receptors to produce a form of physiological antagonism with respect to cytokine release.

The findings in this study support our hypothesis that Δ^9^-THC and NAGly, putatively through GPR18 receptors, regulate morphological and cytokine signaling plasticity in BV-2 microglia. These data add to an emerging profile that emphasizes NAGly as a component of an endogenous system present in the CNS that tightly integrates microglial proliferation, recruitment, and adhesion with neuron-glia interactivity and tissue remodeling. A greater understanding of this system is essential to improving our ability to therapeutically monitor and manage dysregulated microglial activity. 

## AUTHOR CONTRIBUTIONS

Douglas McHugh: cytokine experiments; BV-2 microglia shape categorization; design and coordination of the studies; data interpretation; statistical analyses; and manuscript preparation. Daniel Roskowski: morphology experiments and BV-2 microglia shape categorization and quantification, data interpretation; statistical analyses. Sisi Xie: BV-2 microglia shape categorization and quantification. Heather B. Bradshaw: design and coordination of the studies; data interpretation; statistical analyses; and manuscript preparation. All authors read and approved the final manuscript.

## Conflict of Interest Statement

The authors declare that the research was conducted in the absence of any commercial or financial relationships that could be construed as a potential conflict of interest.
